# Functions of novel Junctional Adhesion Molecule-A (JAM-A) inhibitor in breast cancer cells

**DOI:** 10.1186/1753-6561-9-S1-A46

**Published:** 2015-01-14

**Authors:** XY Lim, K Brennan, AM Hopkins

**Affiliations:** 1 Department of Surgery, Royal College of Surgeons in Ireland, RCSI Education and Research Centre, Beaumont Hospital, Dublin 9, Ireland

## Background

Each year breast cancer is diagnosed in approximately 1 million women worldwide [1]. Our lab has previously linked Junctional Adhesion Molecule A (JAM-A) gene and protein over-expression in breast tumours with an increased risk of metastasis [2,3]. JAM-A loss or inhibition has also been shown to inhibit cell migration and invasion [2], while increasing apoptosis possibly as a result of increased HER2 degradation and downstream Akt signalling [3]. A novel JAM-A inhibitor was designed by our lab which has been found to reduce the migration and proliferation of 4T1 breast cancer cells in vitro, and reduce 4T1 tumour growth in vivo. In this study we sought to determine both the stability and the efficacy of the JAM-A inhibitor on cell proliferation and cell signalling.

## Methods

Cell proliferation was assessed in 4T1 and MCF7-HER2 breast cancer cell lines using the CyQUANT Proliferation Assay. In parallel, changes in the expression and activity of the HER2, ERα and Akt signalling pathways were assessed by western blotting.

## Results & discussion

There was no significant change in cell proliferation between JAM-A inhibitor treated and control treated cells and there was no significant difference in effect between the different JAM-A inhibitor concentrations. This suggests that the JAM-A inhibitor is not stable enough to be stored long-term at -80°C, as it had been prior to the commencement of this study. This conclusion was further supported by western blots showing no significant changes in the expression or activity of HER2, ERα and Akt following treatment with the JAM-A inhibitor.

## Conclusion

The JAM-A inhibitor did not inhibit cell proliferation and had no effect on cell signalling pathways downstream of JAM-A. This highlights the importance of further experiments to determine the precise shelf-life of the inhibitor, and additional chemical alterations may be necessary to increase the stability of the compound.
